# SETD2 is a novel and druggable dependency in IMiD/CELMoD resistant multiple myeloma models

**DOI:** 10.1038/s41408-026-01632-6

**Published:** 2026-10-06

**Authors:** Sarah A. Bird, Marco P. Licciardello, Salomon Morales, Mara Mandelia, Enze Liu, Yakinthi Chrisochoidou, James Smith, Amy Wilson, Jack Cheung, Shannon Martin, Yigen Li, Fernando J. Sialana, Brian A. Walker, Paul A. Clarke, Charlotte Pawlyn

**Affiliations:** 1 The Institute of Cancer Research, London, UK; 2 The Royal Marsden Hospital NHS Foundation Trust, London, UK; 3 Miami Miller School of Medicine, University of Miami, Miami, FL USA

**Keywords:** Myeloma, Targeted therapies

SETD2 is a histone methyltransferase and is the only enzyme known to be capable of trimethylation of histone 3 lysine 36 (H3K36me2 → H3K36me3). SETD2 can also methylate non-histone proteins and is important in transcriptional regulation, RNA splicing, DNA damage repair and B cell development and differentiation [1, 2]. SETD2 inhibition has been shown to reduce the proliferation of Multiple Myeloma (MM) and Diffuse Large B-cell Lymphoma (DLBCL) cell lines and reduce tumour burden in cell line-derived xenograft models [1]. The SETD2 inhibitor EZM0414 was initially granted fast-track FDA designation and investigated in a phase 1 clinical trial in patients with relapsed/refractory MM and DLBCL (NCT05121103), but this study terminated early due to business decisions.

SETD2 inhibition data to date have focused attention on the t(4;14) subgroup of MM patients, as this translocation is a potential biomarker for response. The t(4;14) translocation is found in ~15% of newly diagnosed MM patients and is associated with high-risk disease. The translocation leads to over-expression of Fibroblast Growth Factor Receptor 3 (FGFR3) and Nuclear Receptor Binding SET Domain Protein 2 (NSD2) [3]. NSD2 overexpression leads to increased H3K36 dimethylation and dysregulation of gene expression [4]. Early data suggests that t(4;14) myeloma cell lines (such as H929) may be more sensitive to SETD2 inhibition than other lines and therefore this could be a good drug candidate for patients with this high-risk genetic background [4].

Immunomodulatory agents (IMiDs) and cereblon E3 ligase modulators (CELMoDs) are a cornerstone of MM treatment. They bind to the cereblon (CRBN) component of the CRL4 E3 ubiquitin ligase complex and designate a new set of substrates for proteasomal degradation. Most patients initially respond well to IMiD/CELMoD therapy, but over time become resistant. We generated cell line models of IMiD/CELMoD resistance by treating the IMiD sensitive MM1s and H929 human myeloma cell lines with lenalidomide (Len), pomalidomide (Pom) or iberdomide (Iber) at ~10x their 120-hour GI_50_ for ~12 weeks until resistance was achieved as previously reported [5]. Matched control lines were also generated at the same DMSO concentration. The resistant cell lines were shown to have decreased CRBN protein expression and loss of CRBN function (demonstrated by reduction in Ikaros and Aiolos degradation) [5]. DNA-sequencing analysis of the cell lines demonstrated CRBN mutations in Pom-resistant-MM1s, Pom-resistant-H929 and Iber-resistant-H929 that were predicted to have a high impact on the resultant protein (by Ensembl Variant Effector Predictor) and lead to degraded mRNA or non-functional CRBN [5]. Copy number loss at the CRBN locus was also assessed, with the greatest loss observed in Pom-resistant-H929 and a more modest reduction seen in Len-resistant-H929 [5]. In Len-resistant-MM1s and Iber-resistant-MM1s the drivers of low CRBN expression remain unclear but may be associated with epigenetic modulation or transcriptional repression.

A genome-wide loss-of-function CRISPR screen (Brunello library) was carried out in Iber-resistant-MM1s as previously described [5]. Gene effect scores were calculated using the Chronos algorithm and compared to parental MM1s data from the Broad Institute DepMap portal [5, 6]. Potential new dependencies in the resistant setting (defined as gene effect score <−1 in resistant cells and >−0.5 in parental cells) were identified in 47 genes, including SETD2 [5].

There was no difference in the abundance of SETD2 (Supplementary Fig. 1A) or the global baseline levels of H3K36me2 or H3K36me3 (Supplementary Fig. 1B) in the IMiD/CELMoD-resistant lines compared to the controls on western blot analysis. Two SETD2 inhibitors, EPZ-719 and EZM0414, were then explored in the cell line models. Initial work was performed using EPZ-719 as the clinical compound EZM0414 was not commercially available at the time this work was commenced. However, key experiments were subsequently reproduced with EZM0414. As these drugs are epigenetic modulators 14-day cell growth assays were performed, in keeping with assays described in the literature [1]. Cells were manually counted (Trypan-Blue exclusion assay) and split at day 5 and day 10. EPZ-719 reduced viability to a greater extent in Pom-resistant-MM1s, Iber-resistant-MM1s, Pom-resistant-H929 and Iber-resistant-H929 compared to their controls in a concentration dependent manner (Fig. 1A). A similar trend was also seen in Len-resistant-MM1s and Len-resistant-H929, particularly with 10 µM of EPZ-719 (Fig. 1A). We confirmed a similar effect on viability with EZM0414 in Iber-resistant-MM1s and Iber-resistant-H929 (Fig. 1B). Increased sensitivity to SETD2 inhibition was particularly striking in Pom-resistant-H929 and Iber-resistant-H929 (Fig. 1C); indeed, in these two cell lines a reduction in viability could be seen as early as day 5 at low inhibitor concentrations ( ~ 0.3 μM) (Fig. 1D). An equivalent reduction in the H3K36me3 biomarker for SETD2 inhibition was seen on immunoblotting after EPZ-719 incubation across all cell lines (Supplementary Fig. 1C).

**Fig. 1:**
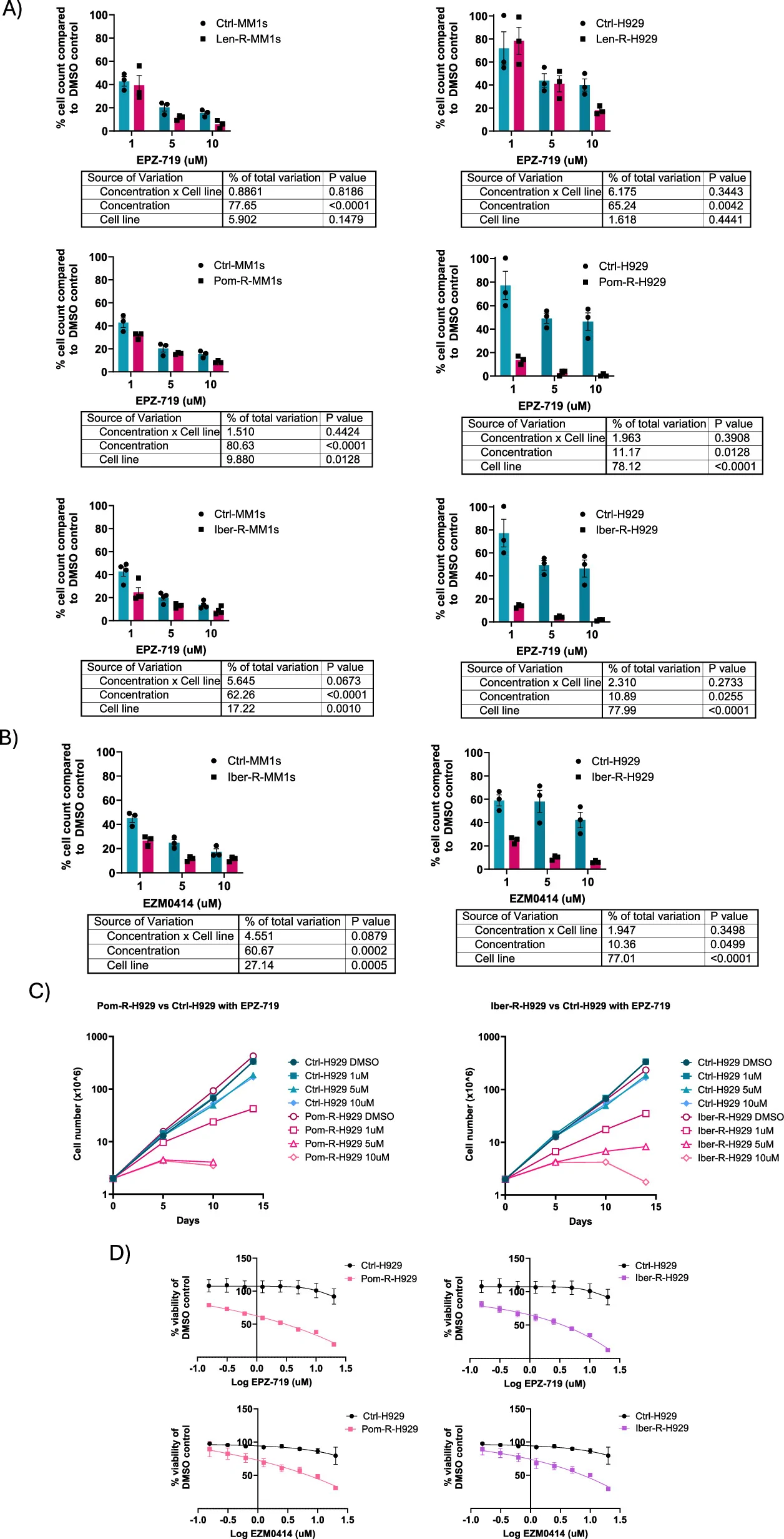
SETD2 inhibitors reduce proliferation to a greater extent in IMiD/CELMoD resistant cell lines compared their control cell lines. **A** Cell proliferation with EPZ-719. Graphs show the calculated total cell count at day 14. EPZ-719 was used at 1, 5 and 10 μM with a DMSO control matching the highest concentration. Individual points represent independent experiments (*n* = 3), the bar represents the mean and error bars show SEM. A two-way ANOVA (with matching of replicates) was performed. **B** Cell proliferation with EZM0414. Graphs show the calculated total cell count at day 14. EZM0414 was used at 1, 5 and 10 μM with a DMSO control matching the highest concentration. Individual points represent independent experiments (*n* = 3), top of bar represents the mean and error bars show SEM. A two-way ANOVA (with matching of replicates) was performed. **C** Time course assays with EPZ-719 over 14 days. Total cell numbers at day 0, 5, 10 and 14 are plotted for each cell line with different concentrations of EPZ-719 (1, 5 and 10 μM) and a DMSO control. Two million cells were seeded per condition on day 0. On days 5 and 10 cells were split, and two million cells per condition were re-seeded with fresh media and drug. The graph shows the total number of cells that would have been present if all cells were kept in culture. **D** Cell proliferation assays over 5 days. A differential reduction in cell viability was seen as early as day 5 in Pom-resistant-H929 and Iber-resistant-H929 with EPZ-719 and EZM0414 in a CellTiter-Blue assay. Graphs show mean and SEM (*n* = 3). The highest concentration of both compounds was 20 µM (with 2-fold dilutions).

The combination of SETD2 inhibition and treatment with iberdomide was then explored. Cells were treated with DMSO or 3 different concentrations of EPZ-719 (1, 5 and 10 μM) for 5 days and then continued to be exposed to the same dose of DMSO/EPZ-719 and varying doses of iberdomide for a further 5 days (Supplementary Fig. 2A). Iberdomide and EPZ-719 were synergistic in Ctrl-Iber-MM1s and were additive, trending towards synergistic, in Ctrl-Iber-H929 (Supplementary Fig. 2B). No synergy was seen in the resistant lines, as without CRBN expression iberdomide had no effect on cell viability (Supplementary Fig. 2B).

There are currently no available clinical datasets to validate enhanced SETD2 *dependency* in IMiD resistant disease and testing SETD2 inhibitors in ex vivo patient myeloma cells is not feasible due to the length of time the cells would need to be kept in culture. However, analysis of the MMRF CoMMpass dataset demonstrated that newly diagnosed MM patients with high expression of SETD2 have significantly shorter progression-free survival (PFS) and overall survival (OS) compared to those with low expression (Supplementary Fig. 3), supporting a role in therapy resistance.

To explore the reasons underpinning greater sensitivity to SETD2 inhibition in the IMiD/CELMoD resistant state (as determined by growth), paired RNA-Seq and ChIP-Seq was performed in Iber-resistant-MM1s and Iber-resistant-H929 (and their matched control lines Ctrl-Iber-MM1s and Ctrl-Iber-H929) (Supplementary Methods 1 & 2). MM1s and H929 cell line pairs were chosen as they have different genetic backgrounds, and we wanted to investigate if there was a shared mechanism for increased sensitivity to SETD2 in the IMiD resistant state in both contexts. Cell lines were exposed to 1 μM of EPZ-719 or DMSO for 3 days prior to harvesting. We selected 3 days of exposure to ensure that altered gene expression detected following treatment resulted from target inhibition rather than secondary effects from decreased viability. At this time point, there were limited changes in cell viability and a global change in H3K36me3 was observed by immunoblotting (Supplementary Fig. 1C).

H3K36me3 is normally an activating mark; therefore, EPZ-719 treatment would be expected to decrease transcript levels of regulated genes. As the IMiD/CELMoD resistant cell lines were more sensitive to SETD2 inhibition than the IMiD/CELMoD sensitive control cell lines, transcripts decreased to a greater extent in Iber-resistant-MM1s and Iber-resistant-H929 (relative to their respective control lines) may indicate the mechanisms of action. 119 transcripts had greater reduced abundance in Iber-resistant-MM1s compared to control and 131 transcripts had greater reduced abundance in Iber-resistant-H929 compared to control after EPZ-719 treatment (Supplementary Fig. 4 and Supplementary Table 1). Three transcripts were commonly differentially downregulated with EPZ-719 vs DMSO in both Iber-resistant-MM1s and Iber-resistant H929 compared to their sensitive control cell lines, Neural Cell Adhesion Molecule 1 (*NCAM1*)/*CD56*, Epithelial Membrane Protein 2 (*EMP2*) and Thymosin Beta 15 A (*TMSB15A*), and therefore might represent a common mechanism of SETD2 dependency in the IMiD resistant state (Fig. 2A). NCAM1/CD56 was of particular interest given its reported importance in MM; where overexpression promotes myeloma cell line growth and viability, while silencing has the opposite effect, reducing growth and increasing apoptotic cell death [7]. We confirmed that NCAM1/CD56 expression was also reduced at the protein level after EPZ-719 treatment (Fig. 2B).

**Fig. 2:**
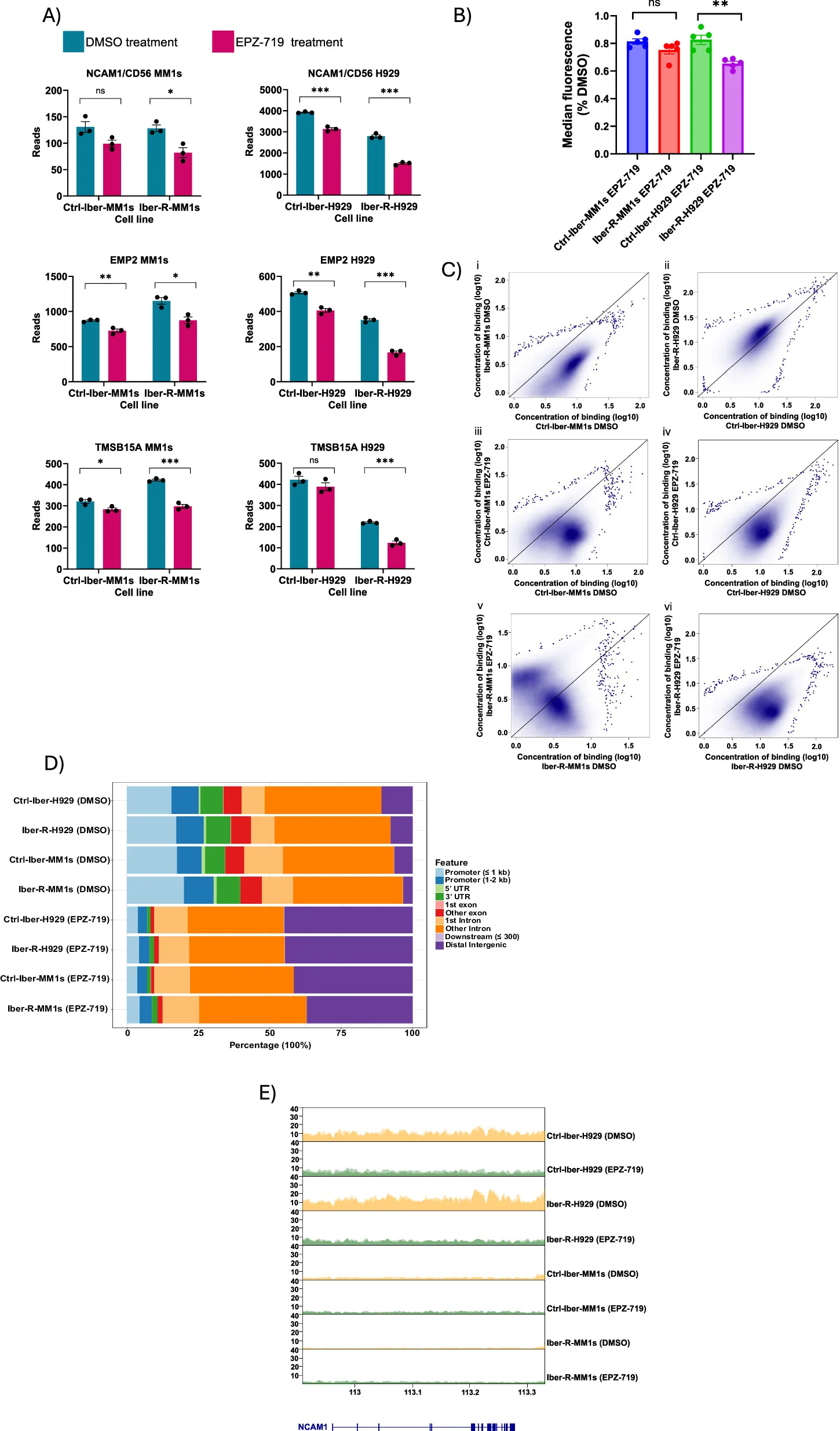
SETD2 inhibition alters RNA expression and H3K36me3 binding. **A** RNA-Seq data was used to identify transcripts that were differentially expressed after EPZ-719 treatment in the same manner in both cell line pairs (Ctrl-Iber-MM1s vs Iber-resistant-MM1s and Ctrl-Iber-H929 vs Iber-resistant-H929). Three transcripts had decreased expression in Iber-resistant-MM1s and Iber-resistant-H929 (defined as ≥ 3 standard deviations of the mean Log2FC for each dataset) but not in their respective control lines. The graphs here show raw counts generated by ht-seq software (the number of reads aligned to each gene). Individual points represent independent biological replicates; the bar represents the mean and error bars represent SEM. Pairwise comparisons were performed using unpaired Student’s t-tests in GraphPad Prism 11. * = *p* < 0.05, ** = *p* < 0.01 and *** = *p* < 0.001. **B** Flow cytometry confirmed a reduction in the expression of NCAM1/CD56 after incubation with 1 µM EPZ-719 for 5 days. Pairwise comparisons were performed using unpaired Student’s t-tests: Ctrl-Iber-H929 to Iber-resistant-H929 and Ctrl-Iber-MM1s to Iber-resistant-MM1s. ** = *p* < 0.01. **C** Density binding plots were used to provide an overview of the ChIP-Seq results. These plots compared the concentration of H3K36me3 binding at sites throughout the genome between pairs of conditions. The axes show concentration of binding (the average number of reads for that binding site) for the two conditions. Overall, this data showed that EPZ-719 reduced H3K36me3 binding across several thousand sites rather than a few selected sites. The raw data was analysed with a ChIP-Seq pipeline built using Nextflow from the Institut Curie NGS/Bioinformatics core facility (https://github.com/bioinfo-pf-curie/ChIP-seq). **D** The relative distribution of the H3K36me3 mark in distinct genic and intergenic regions was then examined in each condition. This stacked bar chart shows the distribution of H3K36me3 binding in each cell line/treatment condition across different genomic features, coloured according to the key provided. **E** Tracks show H3K36me3 ChIP-Seq read densities at genomic regions covering NCAM/CD56 (chromosome 11). The x-axis denotes genomic position (megabases), and the y-axis shows coverage values (normalised to spike-in control and total input control). The Ctrl-Iber-H929 and Iber-resistant-H929 tracks show globally reduced H3K36me3 binding after EPZ-719 treatment. There was less H3K36me3 binding at baseline in the MM1s cell lines and no clear reduction in binding after EPZ-719 treatment. Tracks for the 2 technical replicates of each cell line/treatment condition are overlayed.

A complementary ChIP-Seq experiment was performed to evaluate the effect of EPZ-719 treatment at sites of H3K36me3 binding throughout the genome. Comparison of the baseline pattern of H3K36me3 binding in Ctrl-Iber-MM1s with Iber-resistant-MM1s showed there were genomic sites with decreased binding in the Iber-resistant-MM1s line (Fig. 2Ci). In contrast, the H3K36me3 binding pattern was similar between Ctrl-Iber-H929 and Iber-resistant-H929 (Fig. 2C ii). When treated with EPZ-719 all four of the cell lines showed a broad reduction in H3K36me3 binding across several thousand sites rather than a few selected sites (Fig. 2 C iii-vi). In terms of genomic feature representation, at baseline the H3K36me3 mark was distributed very similarly in the four lines (Fig. 2D). After treatment with EPZ-719 the relative distributions shifted similarly across all lines, with a reduced proportion of binding in the gene promoter, 5’ UTR, 3’ UTR, 1^st^ exon and other exonic regions (Fig. 2D). In Ctrl-Iber-H929 and Iber-resistant-H929, *NCAM1/CD56* had H3K36me3 binding peaks throughout the gene that were reduced globally with EPZ-719 treatment (Fig. 2E). There was less H3K36me3 binding at baseline in the MM1s cell lines and no clear reduction in binding with EPZ-719 treatment (Fig. 2E).

In summary, CRISPR-based screening identified *SETD2* knockout as a novel vulnerability in IMiD/CELMoD resistant cells, and this was validated using two SETD2 inhibitors. This effect was particularly pronounced in the resistant H929 cell lines, which carry the t(4;14) translocation, leading to overexpression of NSD2. The MM1s line does not carry this translocation and so the enhanced sensitivity seen in the H929 resistant lines may be driven by the genetic background, the IMiD/CELMoD resistant state or their interaction.

A ChIP-Seq analysis showed treatment with EPZ-719 in both IMiD resistant cell lines and their respective IMiD sensitive counterparts led to a broad reduction in H3K36me3 binding across several thousand sites. Interestingly, there was also a global reduction in H3K36me3 binding in Iber-resistant-MM1s relative to its control at baseline, a pattern not seen in the H929 pair, suggesting cell line specific epigenetic remodelling upon the acquisition of IMiD resistance.

The broad changes in epigenetic remodelling led to significantly altered expression of several hundred genes in each cell as shown by RNA-Seq analysis. To see if there was a common mechanism in both cell lines, we assessed transcripts that were decreased with SETD2 inhibition to a greater extent in Iber-resistant-MM1s and Iber-resistant-H929 (relative to their respective control lines) and this identified *NCAM1/CD56*, *EMP2* and *TMSB15A*.

NCAM1/CD56 is a glycoprotein aberrantly expressed in >70% of MM patients [8]. NCAM1/CD56 expression is greater in malignant myeloma cells compared to normal plasma cells and the presence of >10% clonal NCAM1/CD56-expressing cells correlates with inferior survival outcomes [8]. Overexpression of *NCAM1/CD56* in low NCAM1/CD56 expressing cells lines (such as MM1s) promotes cell growth and adhesion to bone marrow stromal cells, whilst *NCAM1/CD56* silencing in highly expressing NCAM1/CD56 cell lines (such as H929) leads to the opposite effect [7]. Collectively with our data, this suggests a model where reduced NCAM1/CD56 expression following SETD2 inhibition contributes to cell death, and the effect is greater in the IMiD/CELMoD resistant lines. This differential response is clearer in H929, perhaps because NCAM1/CD56 is expressed ~100 fold greater at baseline (assessed by flow cytometry, data not shown). Significantly, it has been shown that NCAM/CD56 levels can affect cell response to lenalidomide and influence CRBN expression [7] and this putative relationship should be explored further to see if it plays a role in explaining why IMiD resistant cells are so sensitive to SETD2 inhibition.

We propose that decreased NCAM1/CD56 expression may be part of reason for increased sensitivity to SETD2 inhibition in the IMiD resistant state, particularly in the H929 cell line setting, but that downregulation of multiple genes, driven by changes in H3K36me3 across the genome, is likely to be important. In MM1s NCAM1/CD56 expression is low and EPZ-719-induced changes are less pronounced, so although identified as differentially downregulated in our RNA-Seq analysis, this may not be the key driver of differential response in this cell context.

Previous pre-clinical data suggests that the t(4;14) MM subgroup may be more sensitive to SETD2 inhibition, and our data supports this whilst also suggesting that IMiD/CELMoD resistant patients might represent a second target population. An analysis using the MMRF CoMMpass dataset shows newly diagnosed MM patients with high expression of SETD2 have significantly shorter PFS and OS compared to those with low expression, strengthening the argument that this could be a good therapeutic target. Our data reinforces the need for further evaluation of SETD2 inhibition in MM, and supports the inclusion of IMiD resistant patients, rather than the t(4;14) subgroup alone, in further clinical development.

## Supplementary information

Supplementary Material

## Data Availability

The datasets generated during the current study are available from the corresponding author on reasonable request. The RNA-Seq data (GSE348646) and ChIP-Seq (GSE348641) data are deposited in NCBI GEO.
