# Supplementary material for: SETD2 is a novel and druggable dependency in IMiD/CELMoD resistant multiple myeloma models
Source: Blood Cancer J. 2026 Oct 6;16(1):172. doi: 10.1038/s41408-026-01632-6 (PMC13642255; doi:10.1038/s41408-026-01632-6)
Supplement: Supplementary file 1 [file 41408_2026_1632_MOESM1_ESM.docx]

## SETD2 is a novel and druggable dependency in IMiD/CELMoD resistant Multiple Myeloma models

Sarah Bird^1,2^, Marco P Licciardello^1^, Salomon Morales^1^, Mara Mandelia^1^, Enze Liu^3^, Yakinthi Chrisochoidou^1^, James Smith^1^, Amy Wilson^1^, Jack Cheung^1^, Shannon Martin^1^, Yigen Li^1^, Fernando J Sialana^1^, Brian Walker^3^, Paul A Clarke^1^, Charlotte Pawlyn^1,2^

1. The Institute of Cancer Research, London
2. The Royal Marsden Hospital NHS Foundation Trust, London
3. Miami Miller School of Medicine, University of Miami, Florida, US

Contents

[Supplementary Figures	2](#_Toc238037474)

[**Supplementary Figure 1:** Expression of SETD2 in IMiD/CELMoD resistant cell lines	2](#_Toc238037475)

[**Supplementary Figure 2:** Combination assays with EPZ-719 and iberdomide	4](#_Toc238037476)

[**Supplementary Figure 3:** High RNA expression of SETD2 in patient myeloma cells is correlated with shorter PFS and OS	6](#_Toc238037477)

[**Supplementary Figure 4:** Transcriptome changes with EPZ-719 treatment in in the cell line models	7](#_Toc238037478)

[Supplementary Tables	8](#_Toc238037479)

[**Supplementary Table 1:** Transcripts downregulated in the resistant lines compared to their respective controls	8](#_Toc238037480)

[Supplementary Methods	9](#_Toc238037481)

[**Reagents**	9](#_Toc238037482)

[**Supplementary Method 1:** RNA-Seq	9](#_Toc238037483)

[**Supplementary Method 2:** ChIP-Seq	9](#_Toc238037484)

[References:	11](#_Toc238037485)

## Supplementary Figures

### **Supplementary Figure 1:** Expression of SETD2 in IMiD/CELMoD resistant cell lines

A)

B)

C)

A) There was no difference in the normalised abundance of SETD2 in the control and resistant lines on whole proteome analysis (assessed by limma t-test in Phantasus ((1)). Mean and SEM of three biological replicates shown.

B) Immunoblotting showed there was no difference in the baseline expression of H3K36me3 or H3K36me2 in the control and resistant cell lines. Images shown are representative of 2 biological replicates.

C) Immunoblots showed a reduction in global H3K36me3 with 1μM EPZ-719 treatment by day 3. H3K36me2 was unchanged. Images shown are representative of 2 biological replicates.

### **Supplementary Figure 2:** Combination assays with EPZ-719 and iberdomide

A)

B)

A) Cell proliferation assays. Cells were treated with DMSO or EPZ-719 for 5 days and then cultured with the same concentration of DMSO/EPZ-719 and varying doses of iberdomide in an 8-point EC50 CellTiter-Blue viability assay (top concentration 2µM). The y-axis shows the % viability of DMSO control for iberdomide treatment for each cell line and therefore the effect of EPZ-719 as a single agent cannot be inferred from these graphs. However, synergy can be observed; the reduction in viability in Ctrl-Iber-MM1s with the highest dose of iberdomide was ~70% in cells treated with DMSO for 10 days but was ~100% in cells treated with 1μM EPZ-719 for 10 days. Graphs show mean and SEM (n=3).

B) Synergy between EPZ-719 and iberdomide in Ctrl-Iber-MM1s, Iber-resistant-MM1s, Ctrl-Iber-H929 and Iber-resistant-H929 was explored using the ZIP model in SynergyFinder (2). A score <-10 indicates the interaction between two drugs is likely to be antagonistic, a score between -10 and 10 indicates the interaction is likely to be additive, and a score >10 indicates the interaction is likely to be synergistic.

### **Supplementary Figure 3:** High RNA expression of SETD2 in patient myeloma cells is correlated with shorter PFS and OS

A)

B)

An analysis of the relationship between SETD2 RNA expression in the myeloma cells of patients with newly diagnosed MM and outcomes was performed using data from the MMRF CoMMpass study. Pre-treatment bone marrow samples from newly diagnosed patients (n=753) were separated into 4 groups based on their SETD2 expression (quartiles 1-4). Quartile 4 was compared separately to Q3, Q2 and Q1. The p values are derived from Log-rank tests. High RNA expression of SETD2 was significantly correlated with shorter progression free survival (PFS) (A) and overall survival (OS) (B) compared to low expression.

### **Supplementary Figure 4:** Transcriptome changes with EPZ-719 treatment in in the cell line models

A)

B)

A) The log2FCs in the abundance of each transcript with EPZ-719 treatment (compared to DMSO) in Ctrl-Iber-MM1s were plotted on the x-axis and in Iber-resistant-MM1s were plotted on the y-axis. Outliers were defined as values ≥ 3 standard deviations from the mean log2FC for each dataset (indicated on the graph by the pink dotted lines).

B) The log2FCs in the abundance of each transcript with EPZ-719 treatment (compared to DMSO) in Ctrl-Iber-H929 were plotted on the x-axis and in Iber-resistant-H929 were plotted on the y-axis. Outliers were defined as values ≥ 3 standard deviations from the mean log2FC for each dataset (indicated on the graph by the pink dotted lines).

## Supplementary Tables

### **Supplementary Table 1:** Transcripts downregulated in the resistant lines compared to their respective controls

Transcripts that demonstrated decreased expression with EPZ-719 treatment (defined as ≥ 3 standard deviations from the mean log2 fold change for all transcripts per cell line) in the resistant cell line but not its counterpart control line are shown.

| **Transcripts differentially downregulated in Iber-resistant-MM1s (compared to Ctrl-Iber-MM1s)** | | | | | |
| --- | --- | --- | --- | --- | --- |
| ABALON  ACO1  ADGRV1  AHCYL2  AKAP17A  AP2A2  APBA2  APBB2  AQP3  ARHGAP23  ARHGAP27  ARMCX3  ATP2B4  ATP5MC1  BCAN  BLVRB  BSPRY  BST2  C1orf226  CAV1 | CCND1  CD109  CDKL5  CNTN1  CSRP1  CST3  CTTNBP2  CWC25  CXCR3  DAB2IP  DHRS11  DMRTA1  DSE  EMP1  EMP2  EMP3  EPN2  EPOR  ESAM  F12 | FGF2  FMNL2  FXN  GJA1  GPAT3  GPD1L  GPR37  GTF2A1  GUCY1B1  HEYL  HM13  HS3ST1  KLF2  LINC02969  MALAT1  MAMDC2  MGST3  MITF  MORC1  MTUS1 | NALCN  NCAM1  NDNF  NEDD8  NPC2  NRG1  NTRK2  P2RX5  P2RY1  PARD3B  PDXDC1  PECAM1  PLOD2  PLP2  PLXDC2  PRELID1P6  PRR15  PRR15L  PRR34-AS1  PYCR1 | RAB11FIP5  RAB3B  RGPD5  RPL17P18  SEMA6D  SH3PXD2A  SIPA1L2  SLC25A20  SMN1  SMPD3  SNHG9  SPR  SPTLC3  SSR4  ST6GALNAC4  STING1  STT3A  SV2C  SYCP3  SYNM | TALAM1  TBC1D9  TENT5C  TIMP1  TMEM107  TMEM256  TMEM263  TMEM97  TMSB15A  TMTC1  TNFSF15  TOM1L1  TSPAN12  TUBB2B  UBASH3B  VEPH1  VIM-AS1  ZNF740  ZNHIT3 |
| **Transcripts differentially downregulated in Iber-resistant-H929 (compared to Ctrl-Iber-H929)** | | | | | |
| AADAT  ADGRL2  ALOX5  ANLN  APOBEC3B  ASF1B  ASPM  ATP6V0E2-AS1  AURKB  BIRC5  BUB1  BUB1B  C14orf132  C18orf54  CABLES2  CCNA2  CCNB2  CD276  CDC20  CDC25C  CDCA3  CDCA5 | CDK2  CDKN3  CDT1  CENPA  CENPE  CENPF  CENPI  CENPM  CEP55  CIP2A  CIT  CKAP2L  CKS1B  CPNE8  DEPDC1  DEPDC1B  DHFR  DHFRP1  DLGAP5  DMC1  DOCK7  DSCC1 | DTL  E2F2  ECT2  EFR3B  EME1  EMP2  EPB41L3  ESPL1  ETV4  FADS1  FADS2  FANCI  FERMT2  FIGNL1  FOXM1  FREM2  FZD1  GABRQ  GAS2L3  GINS1  GINS2  GTSE1 | GYPC  HROB  HUNK  IQGAP3  KIAA1755  KIF11  KIF14  KIF15  KIF20A  KIF2C  KIF4A  KNL1  LDLRAD3  LINC02321  LOC102724250  LOC105372717  LOC105376781  LOC107985211  LOC124906278  MAFF  MCM10  MELK | MND1  MTBP  MYB  MYBL2  NCAM1  NCAPH  NUF2  OLMALINC  PANK4  PAQR4  PBK  PCLAF  PIMREG  PLK1  PLK4  PLS3  PMP22  POLQ  PRC1  PRR11  PTTG1  RAB27A | RAD54B  SAPCD2  SHCBP1  SIGLEC10-AS1  SIGLEC12  SKA3  SLC37A1  SPAG5  TCF19  TEX15  TGFBR3  THAP10  TICRR  TK1  TMSB15A  TPX2  TROAP  TSPAN18  UBE2C  UPP1  ZNF114 |

## Supplementary Methods

### **Reagents**

*Cell lines*

Cell line identity was confirmed using STR typing (Eurofins) and cells were confirmed mycoplasma negative (MycoStrip, InvivoGen).

*Antibodies*

Immunoblot analysis was performed with antibodies targeting CRBN (Cell Signaling Technology Cat# 71810, RRID:AB_2799810), Ikaros (Cell Signaling Technology Cat# 14859, RRID:AB_2744523), Aiolos (Cell Signaling Technology Cat# 15103, RRID:AB_2744524), GAPDH (Cell Signaling Technology Cat# 5174, RRID:AB_10622025), H3K36me2 (Active Motif Cat# 61019, RRID:AB_2650523), H3K36me3 (Active Motif Cat# 61101, RRID:AB_2615073) and Total H3 (Abcam Cat# ab1791, RRID:AB_302613). Western blots were visualized by Odyssey Fc Imager (LI-COR) using IRDye Infrared Dyes as secondary antibodies.

### **Supplementary Method 1:** RNA-Seq

RNA was extracted from cells using the AllPrep DNA/RNA MiniKit (Qiagen 80204) according to the manufacturer’s instructions. RNA sequencing was carried out at BGI (China). Data processing was performed using Galaxy, with the following pipeline: HISAT2 to align reads to the genome (hg38), htseq-counts to count reads and DESeq2 to make comparisons.

### **Supplementary Method 2:** ChIP-Seq

#### Fixation of cells

Twenty million cells per condition were collected and washed with PBS. The pellet was then re-suspended in 18.8ml of room temperature PBS and 1250µl of freshly opened 16% methanol-free formaldehyde (Thermo Fisher Scientific) added. After careful mixing the sample was rotated for 10 minutes at room temperature. The formaldehyde was then quenched by adding 6ml of cold glycine (Thermo Fisher Scientific) and the cell suspension rotated for 5 minutes at room temperature. Cells were then collected by centrifugation and washed with ice-cold PBS. Cell pellets were snap-frozen in liquid nitrogen and stored at -80^0^C.

#### Processing ChIP-Seq samples

Cells were lysed and the nuclei isolated using buffers containing 1% Triton X-100. Chromatin was then recovered by centrifugation and samples underwent sonication using a Covaris E220 to fragment the chromatin (to ~200-600 base pair fragments). Prior to immunoprecipitation a drosophila spike-in control was added to each sample (Active Motif 53083). Immunoprecipitation was performed using the H3K36me3 antibody (Diagenode C15410192) to capture the histone mark of interest and its bound DNA. Antibody-antigen (and DNA) complexes were affinity captured on magnetic beads, and wash steps performed to remove non-antibody-bound chromatin. A tagmentation approach (which used a Tn5 transposase (Diagenode C01070012) to integrate library preparation) was used and PCR amplification was performed directly from bead-bound immunoprecipitated chromatin. The library was then purified, and size selected using Kappa Pure beads (Roche KK8000).

A total input control was used in this experiment. After the sonication step, a small amount of each small was taken and did not undergo immunoprecipitation. The crosslinks between the protein and DNA were reversed using Proteinase K, and Phenol:Chloroform:Isoamylalcohol used to extract DNA. The same tagmentation approach, library purification and size selection methods were then followed.

The samples were pooled and run on a S1 flow cell at the ICR Genomics Facility. Deconvoluted FASTQ files for each sample were produced.

#### Data analysis

The ChIP analysis was performed using a pipeline built in Nextflow from the Institut Curie NGS/Bioinformatics core facility (https://github.com/bioinfo-pf-curie/ChIP-seq). The outputs of this pipeline included FastQC, BigWig tracks and peak data. Visualisations were created in R.

## References:

1.	Zenkova D, Kamenev V, Sablina R, Artyomov M, Sergushichev A. Phantasus: visual and interactive gene expression analysis. 2018.

2.	Ianevski A, Giri AK, Aittokallio T. SynergyFinder 3.0: an interactive analysis and consensus interpretation of multi-drug synergies across multiple samples. Nucleic Acids Res. 2022;50(W1):W739-W43.

3.	Ben-David U, Siranosian B, Ha G, Tang H, Oren Y, Hinohara K, et al. Genetic and transcriptional evolution alters cancer cell line drug response. Nature. 2018;560(7718):325-30.

4.	Bird SA, Licciardello MP, Sukhtankar A, Morales S, Sialana FJ, Xu Y, et al. IMiD/CELMoD resistance in myeloma is associated with changes in lipid metabolism that may be targetable. Blood Neoplasia. 2026:100281.
